# A distributional approach to obtain adjusted comparisons of proportions of a population at risk

**DOI:** 10.1186/s12982-016-0050-2

**Published:** 2016-06-07

**Authors:** Odile Sauzet, Jürgen Breckenkamp, Theda Borde, Silke Brenne, Matthias David, Oliver Razum, Janet L. Peacock

**Affiliations:** Department of Epidemiology and International Public Health, Bielefeld School of Public Health (BiSPH), Bielefeld University, Bielefeld, Germany; Alice Salomon Hochschule Berlin, University of Applied Sciences, Berlin, Germany; Department of Gynecology, Campus Virchow-Klinikum, Charité Universitätsmedizin Berlin, Berlin, Germany; Division of Health and Social Care Research, King’s College London, London, UK; NIHR Biomedical Research Centre at Guy’s and St Thomas’ NHS Foundation Trust and King’s College London, London, UK

**Keywords:** Dichotomisation, Linear model, Logistic model, Quantile regression

## Abstract

**Background:**

Dichotomisation of continuous data has statistical drawbacks such as loss of power but may be useful in epidemiological research to define high risk individuals.

**Methods:**

We extend a methodology for the presentation of comparison of proportions derived from a comparison of means for a continuous outcome to reflect the relationship between a continuous outcome and covariates in a linear (mixed) model without losing statistical power. The so called “distributional method” is described and using perinatal data for illustration, results from the distributional method are compared to those of logistic regression and to quantile regression for three different outcomes.

**Results:**

Estimates obtained using the distributional method for the comparison of proportions are consistently more precise than those obtained using logistic regression. For one of the three outcomes the estimates obtained from the distributional method and from logistic regression disagreed highlighting that the relationships between outcome and covariate differ conceptually between the two models.

**Conclusion:**

When an outcome follows the required condition of distribution shift between exposure groups, the results of a linear regression model can be followed by the corresponding comparison of proportions at risk. This dual approach provides more precise estimates than logistic regression thus avoiding the drawback of the usual dichotomisation of continuous outcomes.

## Background

Dichotomisation of continuous data is a common practice in medical and epidemiological research and despite being criticised the practice can be justified. One justification is the clinical usefulness of information contained in the dichotomised outcome which is not available in the continuous one. Indeed it is not clear what it means in terms of adverse birth outcomes that the exposure to a specific risk factor reduces the mean birth weight by 100 g. Major limitations of dichotomising continuous outcomes are the loss of power and information but also that power and the magnitude of association depends on the choice of the cut-point [1, 2]. Some authors have investigated the consequence of dichotomising an outcome in the context of regression analyses. Breitling and Brenner [3] have established that dichotomising a log-normal outcome leads to some spurious interactions between two predictor variables. Paige et al. [4] have compared several ways to report weight change as a continuous or categorical outcome and concluded that the significance of predictors included in the model were dependent on the type of outcome. The so called “distributional method” for the dichotomisation of normally distributed outcomes has been introduced for the comparison of two groups [5]. The method is based on the distribution of the continuous outcome and using the delta method [6] provides an estimate for the difference in proportions, risk ratio, or odds ratio with a precision equivalent to the t test performed for the comparison of the means of the continuous outcome. This leads to the dual presentation of a comparison of two means and a corresponding comparison of two proportions. Simulations have shown that under the hypothesis that the data are normally distributed, the distributional method provided estimates of comparison of proportions less biased than the proportions obtained directly from the data [7] and that the sample size required was much lower [5].

However, a non-adjusted comparison of means is rarely the method of choice to analyse data. In this paper we show how the distributional approach can be extended to linear regression models (including mixed models) under the hypothesis of a shift in the distribution between the different levels of exposure and the normality of the model errors. This extended distributional method is illustrated with data from “The Influence of Migration and Acculturation on Pregnancy and Birth Study” to analyse the effect of smoking during pregnancy on adverse birth outcomes. The study was carried out in a 12-month period in 2011/2012 in three maternity hospitals of Berlin, Germany [8]. Reiss et al. [9] have studied the relationship between the risk of smoking in pregnancy and acculturation of the mother using data from this study.

The proportions of low birthweight, small-for-gestational-age and premature birth [10] were each estimated using the distributional method for the dichotomisation of the corresponding continuous outcome. We illustrated the methodology with a discussion of the results of the distributional method applied to the results of linear regression compared to those of a logistic regression which would be the method used for dichotomised outcomes in cross-sectional analysis. We also compare the distributional method to a quantile regression because it is a method commonly used to evaluate effects of an exposure on the values of a continuous outcome but by defining the population at risk by a quantile.

## Methods

### Distributional methods for adjusted comparisons of proportions

The distributional method for the dichotomisation of continuous outcome can be applied to any outcome collected as continuous, for which the continuous data are available with a distribution approximately normal. Moreover, because only the sample sizes, means and standard deviations in each group are necessary for the normal version of the method, it can be applied when only summary statistics are available (for example in meta-analysis [11]).

The distributional method for the dichotomisation of normally distributed outcomes was originally developed for the comparison of two groups [5] under the assumption of equal variance and for unequal variances [7]. The comparison of proportions is obtained from the results of a t test (mean difference). Recent work [12] showed firstly that the distributional method is robust to small deviations from normality and secondly provided a generalised methodology to include data with perturbations to the normal distribution by using the skew-normal distribution.

The distributional approach works using the delta method to obtain the proportions of the population under a threshold value using the parameters of the normal distribution estimated from the data:

1 $$\begin{aligned} p(\overline{X}_n)=\int _{-\infty }^{x_0}f_{N(\overline{X}_n,\sigma ^2)}(t)dt \end{aligned}$$

a large sample standard error [5, 7] is given by

2 $$\begin{aligned} \text {SD}(p(\overline{X}_n))=\frac{s}{\sqrt{n}} f_{N(\overline{x}_n,s^2)}(x_0). \end{aligned}$$

The method assumes that the variance of both groups is known (a correction factor can be used in case of unknown variance ratio) and equal to the pooled maximum likelihood estimator obtained from the data. The estimates for differences in proportions, risk ratios and odds ratios (all three will be called “distributional comparison of proportions” from here on) are obtained by using the maximum likelihood estimators for mean and variance obtained from the data and their measure of uncertainty (standard error) reflect asymptotically the comparison of means (i.e. they have the same precision) by the properties of the delta method.

We now set to generalise the distributional method for use with linear and mixed models. Let *Y* be a random variable with a known threshold which defines the population at risk or needing treatment, e.g. birthweight and the risk posed by having a birthweight under 2500 g. An exposure is defined by a categorical variable *R* with $k+1$ levels, e.g. not smoking during pregnancy, smoking regularly, smoking occasionally. A regression model is obtained to control for confounders or explanatory variables which provides *k* adjusted mean differences by fitting the linear model

3 $$\begin{aligned} Y_i=\beta _0+\beta _{r_i }+\beta X_i+\epsilon _i \end{aligned}$$

where $\epsilon _i$ is the error term for observation *i* following a normal distribution with a mean of 0 and variance $\sigma _e$. The parameter $\beta _r$ represents the adjusted mean differences in outcome values between the rth level of risk and the reference level 0 (no exposure). Also $\beta X_i$ is the matrix notation for the set of parameters for covariates and covariates values other than the exposure. Then using the marginal outcome mean $E(Y|R=r, X)$ obtained from Eq. 3 i.e. obtained from fitting the linear regression model for the $k+1$ levels of exposures, we obtain $k+1$ adjusted distributional probabilities for each level of the exposure $r=0,1,\ldots ,k$, following the same methodology as for Eq. 1

4 $$\begin{aligned} p_r& = {} P(Y<a|R=r,X)=P(\epsilon +E(Y|R=r, X)<a) \\& = {} \Phi \left( \frac{E(Y|R=r, X)-a}{\sigma _e^2}\right) \end{aligned}$$

for a linear regression where $\Phi$ is the cumulative distribution function of the standard normal distribution.

The method can be generalised to mixed models. Here we present a simple random intercept model with two levels

5 $$\begin{aligned} Y_i=\beta _0+\beta _{r_i }+\beta X_i+ \mu _i+\epsilon _i \end{aligned}$$

where $\beta$ is a vector of fixed effects and $\mu$ a random element with zero mean and a variance $\sigma _r^2$ and the error term $\epsilon _i$ with variance $\sigma _e^2$. Then:

6 $$\begin{aligned} p_r& = {} P(Y<a|R=r,X)=P(\mu +\epsilon +E(Y|R=r, X)<a) \\& = {} \Phi \left( \frac{E(Y|R=r, X)-a}{\sigma _e^2+\sigma _r^2} \right) \end{aligned}$$

where $E(Y|R=r, X)$ has been obtained form Eq. 5 for a mixed model and where $\Phi$ is again the standard normal cumulative distribution function.

Assuming a normal distribution for the error, the same methodology applies as in Peacock et al. and Sauzet et al. based on the delta method [5, 7]. This provides an estimate and standard error for $p_r$ which depends on the sample size (i.e. the number of observations with complete data for all covariates) of the exposed subjects at this level, the marginal mean and the common standard deviation for all exposure levels (mean squared error or the squared root of the sum of variances for a mixed model) with the same formulas as in Peacock et al. [5] using the above mentioned data parameters. In the linear case as in Eq. 2:

7 $$\begin{aligned} \text {sd}(p_r)=\frac{s}{\sqrt{n}} \frac{1}{\sqrt{2\pi s^2}}\exp \left( -\frac{(E(Y|R=r, X)-a)^2}{2s^2}\right) \end{aligned}$$

Then an estimate of the value $\Delta$ for the comparison of proportions is obtained with a standard error $se(\Delta$] which reflects asymptotically the precision of the parameter estimate $\beta _r$. This means that if the sample size is large enough $\beta _r/se(\beta _r)$ is very close to the ratio of the estimate for the comparison of proportions by its standard error $\Delta /se(\Delta )$.

### Illustration and comparison with logistic regression and quantile regression

The distributional method for the dichotomisation of continuous outcomes applied to linear regressions was illustrated with data from the study “The Influence of Migration and Acculturation on Pregnancy and Birth”. Data on 6805 pregnancies were collected in a 12 month period (2011/2012) in three maternity hospitals in Berlin, Germany. Here we will consider the relationship between adverse birth outcome and smoking. The dataset contains 7032 birth from 6805 pregnancies, 426 were twins, 24 triplets and 4 quadruplets.

### Outcomes, exposure and covariates

We consider three continuous outcomes with a recognised threshold defining a group at high risk: birthweight (BW), birthweight z-score (see below) and gestational-age. The high risk groups are defined by low birthweight (LBW) babies (BW < 2500 g, ICD-10: P07.1 [10]), small-for-gestational-age (SGA) defined as babies in the lower 10th percentile of the distribution of z-scores (ICD-10: P05.1 [10]), assumed to follow a standard normal distribution and preterm babies with a gestational-age under 37 weeks (ICD-10: P07.03 [10]).

The z-scores were calculated using the algorithm provided by the World Health Organization (WHO) using the mean and standard deviation from the data [13, 14]. The threshold used is the 10th percentile of the standard normal distribution, i.e. ${\mathrm{{a}}}=\,-1.282.$ For the three outcomes, the exposure of interest is smoking during pregnancy. There are three levels in the dataset: non-smoking, smoking regularly, and smoking occasionally. The participants were requested to self report their category without more precision being provided about their meaning. Because of the methodological nature of this work and to keep the presentation simple, the results tables present only the comparisons between non-smokers and regular smokers with the exception of the summary statistics (Table 1).

The model fitted also included the following covariates; for BW: body mass index (BMI) of the mother based on height and weight measurements taken and documented in the course of the first antenatal care visit offered by a medical doctor (on average 9th/10th gestational week), sex and gestational-age; for z-scores: BMI and sex; for gestational-age: BMI and age of mother.

### Comparison

For the comparison of the distributional method of dichotomisation with other regression methods, we fitted three different models to the data. A linear regression model was fitted on the continuous outcome. A linear model assumes a distributional shift (same shape but different means) between the different levels of exposure and a linear relationship between the outcome and the continuous explanatory variables. This means that the relationship is the same in all parts of the distribution. The dual approach consists of presenting alongside adjusted mean differences in outcome between the different levels of exposure and no exposure, the corresponding (marginal) distributional comparison of proportions. For the outcome birthweight we also fitted a mixed model to account for the non independence of siblings in multiple birth [15]. Because the residuals of the model for gestational-age are skewed we used the skew-normal method to obtain the comparison of proportions.

The comparison of proportions obtained from the distributional method was derived from marginal probabilities from the levels of exposure “non-smoking” and “smoke regularly”. Therefore we presented the marginal difference in proportions obtained from the logistic model. Indeed, if $Y_d$ is the dichotomised outcome of interest ($Y_d =0$ if not in the group at risk and $Y_d =1$ if in the group at risk) then using the above notations we have:

$$\begin{aligned} p_r=P(Y_d=1|R=r,X)=P(Y<a|R=r,X) \end{aligned}$$

Therefore a logistic regression and the distributional method are modelling the same object but use two different modelling strategies. The linear relations obtained are not on the same scale.

For a quantile regression [16], no distributional assumptions are needed; moreover a quantile regression model does not assume that the relationship between the outcome and explanatory variables remains constant in all parts of the distribution. It provides the outcome value of a given quantile of the distribution given the covariates. It can be used if none of the methods above can be applied; in particular if no known threshold defines the population at risk. The results of the quantile regression are presented here to show how they differ conceptually from the distributional method for the dichotomisation of continuous outcomes.

For the purpose of comparison, the same covariates were used in each of the three models. The p values presented in the tables are those provided by the software except for the distributional estimates for comparison of proportions. Those were calculated assuming a normal distribution of the estimates as twice the probability that a standard normal random variable has a value greater than the quotient of the estimate by its standard error:

$$\begin{aligned} p\,\textit{value}=2P(X> \beta _r/se(\beta _r))=2\Phi (-\beta _r/se(\beta _r)) \end{aligned}$$

The analyses were performed with Stata 13 (StataCorp. 2013. Stata Statistical Software: Release 13. College Station, TX: StataCorp LP) with the user-written command *reg_distdicho* [17]. Quantile regression parameters plots were obtained using the Stata command grqreg [18].

## Results

Summary statistics for the three continuous and the three dichotomised outcomes are provided for the three levels of smoking in Table 1. The results of the statistical analyses are presented in Tables 2, 3, 4 and 5. Quantile regression parameters are presented for the 10th percentile for BW and z-scores and due to the non-convergence of the model for the 10th percentile, for the 25th percentile for gestational-age.

**Fig. 1:**
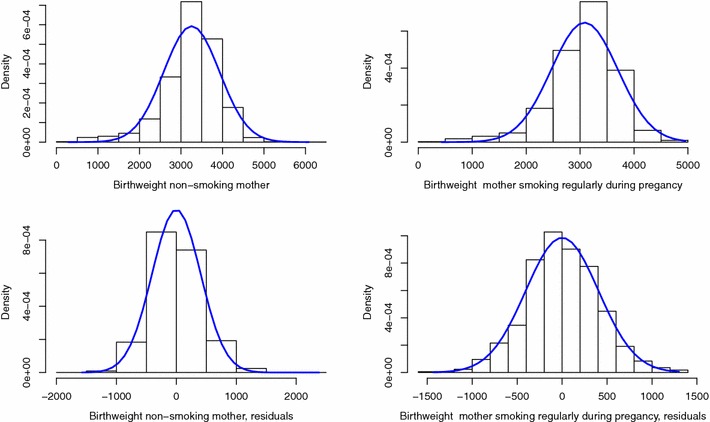
Histograms per exposure group of birthweight and residuals of the linear model adjusting for gestational-age, sex, and BMI of mother at beginning of pregnancy, with normal curve

**Fig. 2:**
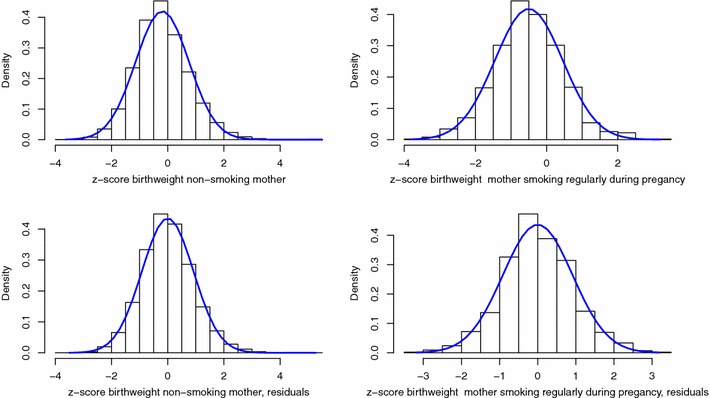
Histograms per exposure group of birthweight z-scores and residuals of the linear model adjusting for sex, and BMI of mother at beginning of pregnancy, with normal curve

**Fig. 3:**
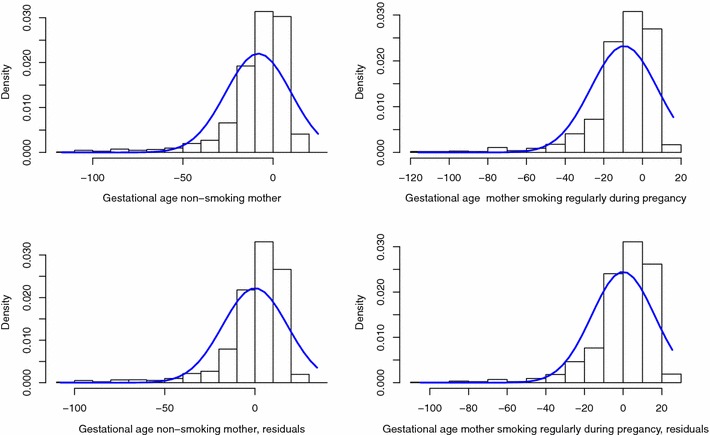
Histograms per exposure group of gestational-age and residuals of the linear model adjusting for sex, age and reported pregnancy at risk, with normal curve

The distributional method for the dichotomisation of continuous outcomes relies on the hypotheses that the residuals of the linear regression are normally distributed and of a distributional shift between the subgroups to be compared (i.e. the subgroups have the same standard deviation). For the three outcomes studied, the density plots (Figs. 1, 2, 3) indicate that the assumption of a distributional shift seems satisfied. It has already been observed that only the term birthweight is normally distributed [19] and as seen in Fig. 1a, b the distribution of birthweight is left skewed. However controlling for gestational-age in the model corrects this and the residuals of the regression model are symmetric, warranting the use of the distributional method for the dichotomisation. We can use this method to obtain an adjusted comparison of the proportion of LBW between mothers who regularly smoked during pregnancy and those who did not. The z-scores and the model residuals for z-scores are all normally distributed (Fig. 2). Gestational-age is not normally distributed and no covariates used in the model explained the tail in the distribution, therefore the residuals also showed a long left tail warranting the use of the skew-normal method.

For low birthweight, marginal values for the difference in proportions provided by the logistic regression [0.022 (0.008)] seem different from the distributional estimates [0.035 (0.005)] (Table 2). But the comparisons of proportions are quite similar between distributional method and logistic regression for small-for-gestational-age (SGA, Table 4) and for pre-terms (Prem, Table 5). However, for the three outcomes there is a difference in precision between the distributional estimates and the results of the logistic regression with greater precision being achieved using the distributional method. For example we can compare the standard errors for the distributional difference in proportions: LBW: 0.005, SGA: 0.010, Prem: 0.004; and the standard errors for the logistic regression marginal difference in proportion: LBW: 0.008, SGA: 0.014, Prem: 0.013 (Tables 2, 4, 5).

If the assumption of distributional shift is satisfied, the relationship between the outcome and covariates remains equal for all parts of the distribution and therefore the effect of smoking on the 10th quantile (or any other quantile) should be similar to the one obtained in the linear regression. Because the quantile regression uses less information and is more volatile, i.e. it is more affected by small changes in observed values than a parametric model, the quantile estimates are less precise than the linear estimates. For BW and SGA z-scores of the quantile estimates are reasonably close to the linear ones. But the standard errors are between 72 and 77 % larger for quantile than for linear regression.

**Fig. 4:**
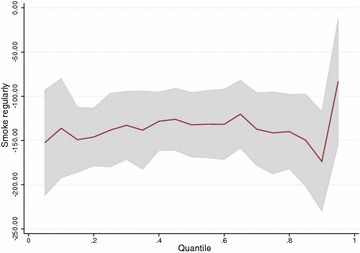
Quantile regression parameters per quantile for variable smoking regularly versus non-smoking (reference) for the outcome birthweight (g)

**Fig. 5:**
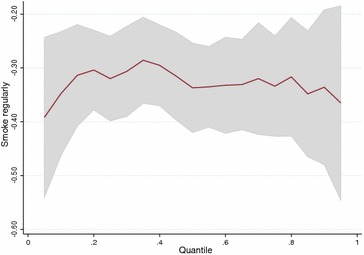
Quantile regression parameters per quantile for variable smoking regularly versus non-smoking (reference) for the outcome birthweight z-score

**Fig. 6:**
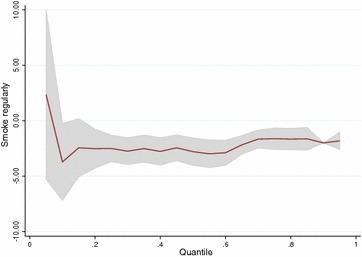
Quantile regression parameters per quantile for variable smoking regularly versus non-smoking (reference) for the outcome gestational-age (days)

For gestational-age the estimates for the group effect on first quartile $(-2.50)$ disagree from the linear model $(-1.81)$ (Table 5). This indicates that the effect of smoking might be different in the lower part of the distribution than in the upper parts. We obtained plots of the quantile regression parameters for smoking regularly for all quantiles between 0.1 and 0.9 for the three outcomes (Figs. 4, 5, 6). They show that the effect of smoking regularly on birthweight and birthweight z-scores remains constant for all quantiles. For gestational-age the parameters are inconsistent because of the small effects but there is a clear indication that the effect of smoking regularly seems stronger on the lower half of the distribution.

In order to illustrate the use of the distributional method for mixed models we fitted a random intercept model to the birthweight outcome to control for multiple births (Table 3). This led to a difference of 5 g in the adjusted difference in birthweight between non-smokers and regular smokers which only marginally affected the comparison of proportion of LBW babies compared to ignoring the non-independence of siblings.

## Discussion

The practice of dichotomisation has been strongly criticised due to the clear loss of information and because dichotomisation fails to show the true relationship between an outcome and its possible predictors as shown by Breitling et al. [3]. The distributional method for the dichotomisation of continuous outcomes provides a solution to some of these difficulties. We have shown in this paper how this method could be applied to the results of a linear regression. The precision or the significance of a statistical test will not depend on the choice of threshold contrary to when the data are directly dichotomised as pointed out by Altman et al. [2] because the only statistical test performed is based on the comparison of mean values of the continuous outcome. The distributional estimates obtained from a linear regression reflect the same relationship between covariates as with the continuous outcome. Previous work has been concerned of the effect of dichotomisation in individual studies when meta-analyses are performed. Liu et al. [20] have recently proposed a method for meta-analysis with heterogeneous outcomes using summary statistics and Ofuya et al. [11] have shown how the distributional method for the dichotomisation of continuous outcomes, because it could be applied using only summary statistics, could be useful when the study outcomes were heterogeneous. However, the distributional method applied to adjusted means required the knowledge of the marginal means for the various levels of exposure which are not usually provided in study reports and therefore limits the use of the method presented in this article in meta-analyses.

When the error term is not normally distributed and this cannot be corrected by adding the relevant explanatory variable (e.g. gestational-age to explain the tail in the distribution of birthweights) then there is an alternative distributional method which can be used for perturbation to the normal distribution [12] using the skew-normal distribution as we did for gestational-age. If the standard deviation is not the same in different levels of exposure, a correction factor can be applied [7].

### Comparison with other regression models

We have compared the results obtained by using the distributional method for the dichotomisation of continuous outcomes to other existing modelling approaches using the same covariates.

We wanted to show how the distributional method compares to other regression models where the effect of a risk factor in predicting the group at risk can be estimated. The logistic regression model is the ‘data’ equivalent of the distributional method for dichotomisation applied to linear regression, i.e. it is the analogue of the use of the data in estimating the proportion at risk (i.e. number at risk divided by the total) as opposed to estimating this proportion from the distribution of residuals and regression parameters estimates. Because the distributional method gives results based on marginal effects, we compared results of the distributional method with results of the logistic regression based on the marginal proportions. Moreover it has been recommended that researchers provide results of a logistic regression based on marginal effects [22] to improve the comparability across studies. The results of the logistic regression were less precise than the ones using the distributional method because the later has an equivalent precision to a linear regression and uses all of the data, unlike logistic regression which uses the dichotomous outcome corresponding to what has been observed in terms of sample size in [5] and using simulation in [7] in previous work. Hence, while the distributional method and logistic regression are modelling the same object, the estimates are obtained using two different modelling strategies which can lead to differences in estimates for the comparison of proportions. Moreover, and this is the problem the distributional method aims to solve, proportions estimated from the data (logistic regression) can be volatile if the sample size is small. In regression models with several covariates, some subgroups can be small leading to unstable estimated probabilities that are strongly affected by small differences in the data. Logistic regression might be the only alternative when model assumptions for the distributional method for dichotomisation are not satisfied and it can also be applied if the continuous outcome has been previously dichotomised and is no more available.

Quantile regression is a useful tool to estimate the effect of a risk factor as a predictor of being in a group at risk but it is not a modelling tool for proportions. The group at risk can only be identified as being included in a particular tail of the distribution, e.g. 10th percentile. If a threshold exists, its value can be compared to the values of the percentile modelled. If the relationship between covariates is dependent on which part of the distribution the outcome is in then the assumptions of a linear model are not satisfied. In this situation a linear model would be misspecified and a quantile regression is an alternative which can provide useful information. Obtaining a plot of quantile regression parameters per quantile is also a useful exploratory tool to assess the hypothesis of distribution shift.

### Limitations and future work

A limitation of the methodology is that it can be only applied if the hypotheses underlying the linear model are satisfied, i.e. the error term is normally distributed and the standard error is constant over all subgroups (distribution shift). If these assumptions are strongly violated the results might not be reliable. In this work we have not performed new simulations for the comparison between the various methods due to its illustrative nature. However, evidence have been obtained in previous work [7] for unadjusted differences where the method was validated and compared to a direct comparison of proportions. Nonetheless it would be interesting to obtain a systematic power comparison between logistic regression and distributional method for the dichotomisation of continuous outcomes for various types and numbers of covariates. This goes beyond the scope of this paper and should be the topic of further work.

The present work considered only categorical exposure levels. A review of the practice in the literature [21] showed that in 86 % of the reviewed papers, continuous exposures were categorised. Developing the method for continuous exposure is the object of further work. Also the extension of the method to other distributions for the residuals of the linear regression have not yet been addressed.

The misclassification of dichotomised variables is a consequence of measurement errors in the continuous variable. It has been shown that a measurement error in the outcome (unlike for covariates) can be ignored in a linear regression because the additive modelling of the error does not affect the relationship with covariates. Only the power and precision are affected by adding an error term [23]. However this is not the case for the dichotomised outcome because the misclassification into the wrong category has no equivalent additive model. In that case misclassification is a source of bias.

This means that the presence of an independent measurement error in the continuous outcome (i.e. independent of the outcome and its covariates) does not affect the linear relationship between covariates and outcome. This is the basis of the estimation of the distributional comparison of proportions. Therefore those estimates are free of misclassification bias whereas those obtained from logistic regression are not. However this issue should be further investigated. It has been argued by Shentu et al. [24] that in the presence of more complex forms of systematic error in the continuous outcome, a dichotomised outcome may perform better than the continuous one.

### Conclusion

The distributional method for the dichotomisation of continuous outcomes applied to results of a linear regression is a useful alternative to logistic regression when a group at risk is defined by an agreed threshold and when the model conditions apply and the continuous outcome is available. The method has the advantages of offering more stable estimates, being more powerful and less sensitive to misclassification than logistic regression.

**Table 1:** Summary statistics of outcomes per smoking category: birthweight (BW) and low birthweight (birthweight $\le$ 2500 g), birthweight z-score (adjusted for gestational-age) and small-for gestational-age (z-score < $- 1.282$), gestational-age (GA) and preterm (GA < 37)

	N^a^	Mean (SD)	LBW (%)	Mean (SD)	SGA (%)	Mean (SD)	Pretem (%)
		BW (g)		z-score		GA (weeks)	
Smoke regularly	838	3089 (608)	13.3	−0.51 (0.95)	19.6	38.7 (2.5)	14.0
Smoke occasionally	343	3171 (656)	9.8	−0.35 (0.95)	16.9	38.7 (2.6)	11.8
Don’t smoke	4836	3263 (670)	10.4	−0.19 (0.95)	11.0	41.1 (2.5)	12.3

^a^Only data with BMI available

**Table 2:** Birthweight (g) and risk of low birthweight (birthweight < 2500 g) of babies of mother smoking regularly versus not smoking (reference)

${\mathrm{{N}}}=6002$	Estimate (SE)	95 % confidence interval	*p* value
Adjusted mean difference (linear regression)^a^	−145.7 (15.2)	[−175.5, −115.9]	<0.0001
Adjusted distributional comparison of proportions^a^			
Marginal difference in proportions	0.035 (0.005)	[0.026, 0.043]	<0.0001
Marginal risk ratio	2.13 (0.16)	[1.94, 2.46]	<0.0001
Marginal odds ratio	2.20 (0.18)	[1.89, 2.58]	<0.0001
Logistic regression^a^			
Odds ratio	1.61 (0.27)	[1.16, 2.24]	0.004
Marginal difference in proportions	0.022 (0.008)	[0.006, 0.037]	
Quantile regression for the 10th percentile^a^	−152.9 (26.1)	[−204.2, −101.7]	<0.0001

^a^Adjusted for gestational-age, sex, and BMI of mother at beginning of pregnancy

**Table 3:** Birthweight (g) and risk of low birthweight (birthweight < 2500 g) of babies of mother smoking regularly versus not smoking (reference) with a mixed model to take multiple births into account

${\mathrm{{N}}}=6002$	Estimate (SE)	95 % confidence interval	*p* value
Adjusted mean difference (linear regression)^a^	−150.3 (15.3)	[−180.4, −120.3]	<0.0001
Adjusted distributional comparison of proportions^a^			
Marginal difference in proportions	0.035 (0.005)	[0.026, 0.044]	<0.0001
Marginal risk ratio	2.19 (0.075)	[1.89, 2.53]	<0.0001
Marginal odds ratio	2.27 (0.080)	[1.95, 2.66]	<0.0001
Logistic regression^a^			
Odds ratio	2.07 (0.50)	[1.29, 3.31]	0.002

^a^Adjusted for gestational-age, sex, and BMI of mother at beginning of pregnancy

**Table 4:** Birthweight z-score^a^ and risk of small for-gestational-age^a^ of babies of mother smoking regularly versus not smoking (reference)

${\mathrm{{N}}}=6002$	Estimate (SE)	95 % confidence interval	*p* value
Adjusted mean difference (linear regression)^b^	−0.336 (0.034)	[−0.403, −0.268]	<0.0001
Adjusted distributional comparison of proportions^a^			
Marginal difference in proportions	0.088 (0.010)	[0.068, 0.108]	<0.0001
Marginal risk ratio	1.76 (0.054)	[1.58, 1.95]	<0.0001
Marginal odds ratio	1.95 (0.066)	[1.71, 2.22]	<0.0001
Logistic regression^a^			
Odds ratio	2.10 (0.21)	[1.72, 2.54]	<0.0001
Marginal difference in proportions	0.092 (0.014)	[0.064, 0.120]	
Quantile regression for the 10th percentile^a^	−0.348 (0.060)	[−0.468, −0.230]	<0.0001

^a^Gestational-age adjusted birthweight z-score, threshold for small for gestational-age: $-1.282$

^b^Adjusted for sex, and BMI of mother at beginning of pregnancy

**Table 5:** gestational-age (days) and risk of premature birth (gestational-age lower than 37 weeks) of babies of mother smoking regularly versus not smoking (reference)

${\mathrm{{N}}}=6002$	Estimate (SE)	95 % confidence interval	*p* value
Adjusted mean difference (linear regression)^a^	−1.81 (0.67)	[−3.31, −0.050]	0.007
Adjusted distributional comparison of proportions^a^			
Marginal difference in proportions	0.023 (0.004)	[0.014, 0.033]	^b^
Marginal risk ratio	1.11 (0.021)	[1.07, 1.16]	^b^
Marginal odds ratio	1.14 (0.027)	[1.08, 1.21]	^b^
Logistic regression^a^			
Odds ratio	1.26 (0.13)	[1.01, 1.56]	0.039
Marginal difference in proportions	0.025 (0.013)	[−0.001, 0.051]	
Quantile regression for the 25th percentile^a^	−2.50 (0.68)	[−3.83, −1.18]	<0.0001

^a^Adjusted for BMI and age of mother at beginning of pregnancy

^b^The skew normal dichotomisation does not reflect the linear regression model in its precision
